# Immunological Analysis of Nodavirus Capsid Displaying the Domain III of Japanese Encephalitis Virus Envelope Protein

**DOI:** 10.3390/pharmaceutics13111826

**Published:** 2021-11-01

**Authors:** Kiven Kumar, Hui Kian Ong, Wen Siang Tan, Siti Suri Arshad, Kok Lian Ho

**Affiliations:** 1 Department of Pathology, Faculty of Medicine and Health Sciences, Universiti Putra Malaysia (UPM), Serdang 43400, Selangor, Malaysia; kivenkumar@yahoo.com (K.K.); onghk1991@gmail.com (H.K.O.); 2 Department of Microbiology, Faculty of Biotechnology and Biomolecular Sciences, Universiti Putra Malaysia (UPM), Serdang 43400, Selangor, Malaysia; wstan@upm.edu.my; 3 Laboratory of Vaccines and Biomolecules, Institute of Bioscience, Universiti Putra Malaysia (UPM), Serdang 43400, Selangor, Malaysia; 4 Department of Veterinary Pathology and Microbiology, Faculty of Veterinary Medicine, Universiti Putra Malaysia (UPM), Serdang 43400, Selangor, Malaysia; suri@upm.edu.my

**Keywords:** Japanese encephalitis vaccine, *Macrobrachium rosenbergii* nodavirus, virus-like particles (VLP), domain III, cytokines, cytotoxic T-lymphocytes

## Abstract

Japanese encephalitis virus (JEV) is the pathogen that causes Japanese encephalitis (JE) in humans and horses. Lethality of the virus was reported to be between 20–30%, of which, 30–50% of the JE survivors develop neurological and psychiatric sequelae. Attributed to the low effectiveness of current therapeutic approaches against JEV, vaccination remains the only effective approach to prevent the viral infection. Currently, live-attenuated and chimeric-live vaccines are widely used worldwide but these vaccines pose a risk of virulence restoration. Therefore, continuing development of JE vaccines with higher safety profiles and better protective efficacies is urgently needed. In this study, the *Macrobrachium rosenbergii* nodavirus (*Mr*NV) capsid protein (CP) fused with the domain III of JEV envelope protein (JEV-DIII) was produced in *Escherichia coli*. The fusion protein (*Mr*NV-CP^JEV-DIII^) assembled into virus-like particles (VLPs) with a diameter of approximately 18 nm. The BALB/c mice injected with the VLPs alone or in the presence of alum successfully elicited the production of anti-JEV-DIII antibody, with titers significantly higher than that in mice immunized with IMOJEV, a commercially available vaccine. Immunophenotyping showed that the *Mr*NV-CP^JEV-DIII^ supplemented with alum triggered proliferation of cytotoxic T-lymphocytes, macrophages, and natural killer (NK) cells. Additionally, cytokine profiles of the immunized mice revealed activities of cytotoxic T-lymphocytes, macrophages, and NK cells, indicating the activation of adaptive cellular and innate immune responses mediated by *Mr*NV-CP^JEV-DIII^ VLPs. Induction of innate, humoral, and cellular immune responses by the *Mr*NV-CP^JEV-DIII^ VLPs suggest that the chimeric protein is a promising JEV vaccine candidate.

## 1. Introduction

The Japanese encephalitis virus (JEV) is a vector-borne zoonotic virus responsible for encephalitis in domestic animals, including humans. JEV is detected in most of Asian and Oceania countries, including China, Japan, Taiwan, South Korea, Vietnam, Thailand, India, Sri Lanka, Cambodia, Indonesia, Philippines, Australia, and Malaysia [1,2]. Recently, JEV was also detected in African and European countries [3,4,5]. Aquatic wading birds have been identified as reservoirs, while pigs and bats represent the virus-amplifying hosts, whereas the dead-end hosts comprise humans and equid. Humans can be infected by JEV via bites of the mosquito, *Culex tritaeniorhynchus*. Japanese encephalitis (JE) patients develop early mild symptoms such as fever, diarrhea, rigor, and headache, followed by more severe symptoms, including sudden onset of headache, neck stiffness, disorientation, seizures, convulsion, coma, spastic paralysis, and eventually death [6]. The fatality rate of JEV infection was reported to be between 20–30%, of which, 30–50% of the survivors were reported to suffer from permanent neuropsychiatric sequelae [7,8]. Several studies demonstrated that JEV could infect all age groups, but a higher mortality rate (over 30%) was reported among children than other age groups. Approximately 3 billion people who live in JEV endemic countries are at risk of JEV infection [9].

JEV is a single-stranded positive-sense RNA (+ssRNA) virus with an RNA genome (~10 kb) that encodes three structural proteins; the envelope (E), capsid (C) and precursor membrane (prM) proteins, as well as seven non-structural (NS) proteins designated as NS1, NS2A/B, NS3, NS4A/B, and NS5 [10]. The E protein comprises three major domains known as Domains I (DI), II (DII), and III (DIII). The DIII of JEV E protein (JEV-DIII) plays an important role in antibody recognition. Numerous neutralizing epitopes found in JEV-DIII were shown to induce protective neutralizing-antibody response in hosts [11].

To date, an antiviral drug against JEV infection remains unavailable. Therefore, vaccination represents the most effective approach for virus prevention and control. Widely used commercial JEV vaccines comprise live-attenuated and chimeric-live vaccines (ChimeriVax-JE) [12]. However, some major drawbacks have been reported, particularly the risk of conversion to virulence strains in live-attenuated vaccines. Monath et al. [13] reported that a single point mutation in the envelope protein (Met279Lys) produced a neurovirulence strain in fetal monkey liver cells and suckling mice, indicating the hidden risks of using live-attenuated vaccines. Hence, continuing development of JEV vaccines is required to enhance the safety and effectiveness of the vaccines.

*Macrobrachium rosenbergii* nodavirus (*Mr*NV) is the causative agent responsible for white-tail disease in giant freshwater prawns [14]. *Mr*NV is a non-enveloped aquatic virus containing a bipartite genome of +ssRNA. The larger RNA molecule (~3.1 kb) encodes the RNA-dependent RNA polymerase (RdRp) and B2, while the smaller counterpart (~1.2 kb) encodes the virus capsid protein (CP). The *Mr*NV-CP expressed in *Escherichia coli* has been shown to self-assemble into virus-like particles (VLPs) [15]. These VLPs have been manipulated as nanocarriers for intracellular delivery of drugs, DNA, and RNA molecules [16,17,18]. Recently, the VLPs were modified genetically to display foreign epitopes for vaccine developments [19,20,21,22]. VLPs are assembled from multiple copies of viral structural proteins, and these particles morphologically mimic native viruses, but they are neither replicative nor infectious due to the lack of genetic materials. Viral vectors, on the other hand, are recombinant viruses which function as carriers that deliver a coding sequence of a foreign epitope intracellularly by natural infection [23]. Upon delivery of the coding sequence into the target cells, the epitope will be expressed using the host cell protein expression machinery. The *Mr*NV-CP-based VLPs displaying foreign epitopes have been shown to induce high levels of antibodies against the displayed epitopes in BALB/c mice and increase the survival of BALB/c mice challenged with influenza A viruses [20]. In this study, the full-length *Mr*NV-CP was modified to display the DIII of JEV E-protein at its C-terminal end. The fusion protein (*Mr*NV-CP^JEV-DIII^) expressed in *E. coli* self-assembled into VLPs. The immunogenicity of *Mr*NV-CP^JEV-DIII^ VLPs was investigated using BALB/c mice. The results demonstrated that the BALB/c mice immunized with *Mr*NV-CP^JEV-DIII^ VLPs produced significant levels of innate, humoral, and cellular immune responses. These findings suggest that *Mr*NV-CP^JEV-DIII^ self-assembled into VLPs is a potential JEV vaccine candidate.

## 2. Materials and Methods

### 2.1. Construction of Recombinant Plasmids

The reference strain, JEV Nakayama strain [24], was used in this study. The JEV-DIII coding sequence was amplified using one-step reverse-transcription polymerase chain reaction (RT-PCR) with *Taq* polymerase (Promega, Madison, WI, USA) and a pair of primers (forward primer: 5′GCCACCTAAAATGCAGGCTGA3′ and reverse primer: 5′TTTGAGCTCCCTTCAAAGTCG3′, 10 µM each) in an Eppendorf Mastercycler (Eppendorf, Hamburg, Germany). The RT-PCR was performed with a 45 min reverse transcription at 45 °C, followed by an initial denaturation at 95 °C for 1 min, 40 cycles of denaturation at 94 °C for 30 s, annealing at 52 °C for 1 min, extension at 72 °C for 2 min, and completed with a final extension at 72 °C for 5 min. The amplified coding region was cloned into the pTZ57R/T vector using the InsTAclone PCR Cloning Kit (Thermo Fisher Scientific, Waltham, MA, USA) according to the manufacturer’s protocol. The positive plasmid was extracted by using the QIAprep Spin Miniprep Kit (Qiagen, Hilden, Germany). The coding fragment of JEV-DIII flanked by *Eco*RI and *Hin*dIII restriction sites at its 5′ and 3′ ends, respectively, was amplified with a pair of primers (forward primer: 5′GGGGGGAATTCGGTGGTGGTTGGAGCCACCTAAAATGCAGGCTGAGAATGG3′ and reverse primer: 5′GGGGGAAGCTTGGGTTTGAGCTCCCTTCAAAGTCGTTGAAAAGG’3; *Eco*RI and *Hin*dIII restriction sites are underlined), and Velocity DNA polymerase (Bioline, London, UK). The PCR was performed with an initial denaturation at 98 °C for 5 min, followed by 35 cycles of denaturation at 98 °C for 30 s, annealing at 66 °C for 1 min, extension at 72 °C for 10 min, and a final extension at 72 °C for 10 min. The amplicon was purified by using the QIAquick PCR Purification Kit (Qiagen, Hilden, Germany) and Gel Cleanup Kit (Qiagen, Hilden, Germany). The pTrcHis-TARNA2, which contains the coding sequence of the full-length *Mr*NV-CP [15], and the purified amplicon were digested with *Eco*RI and *Hin*dIII (Thermo Scientific, Waltham, MA, USA). The digested pTrcHis-TARNA2 plasmid and PCR-amplified JEV-DIII were ligated with T4 DNA ligase (Promega, Madison, WI, USA) by incubating the ligation mixture overnight at 4 °C. The ligated plasmid (pTrcHis-TARNA2-JEVDIII) was transformed into *E. coli* TOP10 competent cells (Thermo Fisher Scientific, Waltham, MA, USA) using the heat shock transformation method. The nucleotide sequence of the insert in the recombinant plasmid was verified by nucleotide sequencing.

For protein expression, the nucleotide sequence encoding the JEV-DIII was cloned into pTrcHis2-TOPO vector. The JEV-DIII coding sequence with restriction enzyme cutting sites was amplified with a pair of primers (forward primer: 5′AAATTTACCATGGCCCTTATGGACAAACTGGCTCTGAAAGGC3′ and reverse primer: 5′TTCGAATTCGCCCCTGCCCAGCGTGCTTCCAGCCTTGTGCCAATGGTG3′; the *Nco*I and *Eco*RI restriction sites are underlined). The PCR was performed with an initial denaturation at 98 °C for 5 min, followed by 35 cycles of 98 °C for 30 s of denaturation, 70 °C for 1 min of annealing, and an extension of 72 °C for 1 min. The final extension was performed at 72 °C for 10 min. The PCR product was purified, ligated into the digested pTrcHis2-TOPO vector, and introduced into *E. coli* TOP10 competent cells, as described above.

### 2.2. Protein Expression and Purification

The expression protocol of the chimeric protein was adapted from previous studies [15,21]. In brief, bacterial cells carrying the plasmid pTrcHis-TARNA2-JEVDIII were induced by 1 mM isopropyl β-D-1-thiogalactopyranoside (IPTG, Bio Basic, Markham, ON, Canada) to express the recombinant protein at 30 °C for 5 h. Bacterial cells were harvested by centrifugation at 8000× *g* for 5 min. The cell pellets were resuspended in a 10 mL lysis buffer (25 mM HEPES, 500 mM NaCl, pH 7.4). The cell suspension was added with MgCl_2_ (4 mM), lysozyme (0.2 mg/mL), DNase 1 (20 µg/mL), and phenylmethylsulfonyl fluoride (PMSF, 2 mM), and incubated for 2 h on a rotator at room temperature. The cells were then sonicated at 30 MHz for 10 s for 15 cycles, with 20 s intervals for cooling. The cell lysates were centrifuged at 13,000× *g* for 20 min. The crude lysate was collected and filtered through a 0.45 µm syringe filter (Pall Corporation, Ann Arbor, MI, USA) prior to purification with HisTrap HP 1mL column (GE Healthcare, Uppsala, Sweden) pre-equilibrated with washing buffer A (25 mM HEPES, 500 mM NaCl, pH 7.4). The sample was loaded onto the column and washed with 10 column volumes (CV) of washing buffer B (25 mM HEPES, 500 mM NaCl, 50 mM imidazole, pH 7.4). Bound proteins were then eluted from the column by 6 CV of elution buffer (25 mM HEPES, 500 mM NaCl, 100 mM imidazole, pH 7.4). The purified protein was then analyzed by SDS-polyacrylamide gel electrophoresis (SDS-PAGE) and western blotting.

### 2.3. SDS-Polyacrylamide Gel Electrophoresis and Western Blotting

The purified protein was mixed with 6× SDS-PAGE loading buffer [100 mM Tris-HCl, pH 6.8, 0.2% (*w*/*v*) bromophenol blue, 4% (*w*/*v*) SDS, 20% (*v*/*v*) glycerol and 5% (*v*/*v*) of β-mercaptoethanol] and boiled at 95 °C for 10 min. The denatured samples were loaded onto 12% (*w*/*v*) SDS-polyacrylamide gel and electrophoresed at 16 mA for 80 min. The SDS-polyacrylamide gel was stained with staining solution [0.1% (*w*/*v*) Coomassie brilliant blue R-250, 10% (*v*/*v*) acetic acid and 40% (*v*/*v*) methanol] and de-stained with de-staining buffer [10% (*v*/*v*) acetic acid and 30% (*v*/*v*) methanol].

In western blotting, the nitrocellulose membrane was pre-soaked in 1× transfer buffer [20% (*v*/*v*) methanol, 192 mM glycine and 25 mM Tris-HCl, pH 8.3] for 5 min. The separated proteins on an electrophoresed SDS-PAGE gel were electro-transferred to a nitrocellulose membrane using a semi-dry protein trans-blotter (Bio-Rad, Hercules, CA, USA). The nitrocellulose membrane was blocked with 10% (*w*/*v*) skimmed milk (Anlene, Auckland, New Zealand) at room temperature for 1 h. The primary antibody [anti-His monoclonal antibody (mAb) (MERCK, Kenilworth, UK) with 1:3000 dilution in TBS (50 mM Tris-HCl, 150 mM NaCl, pH 7.4) or anti-JEV-DIII mAb (MyBioSource, San Diego, CA, USA) with 1:2500 dilution in TBS] was added, and incubated overnight at 4 °C. The nitrocellulose membrane was then washed 4× with TBST buffer [TBS containing 0.01% (*v*/*v*) Tween-20] followed by addition of the anti-mouse secondary antibody conjugated to alkaline phosphatase (goat anti-mouse IgG-HRP; 1:5000 dilution in TBS; Santa Cruz Biotechnology, Dallas, TX, USA). The membrane was incubated for 1 h at room temperature and washed again as described above. Lastly, a premixed 5-bromo-4-chloro-3-indolyl phosphate (BCIP) and nitro blue tetrazolium (NBT) solution (Santa Cruz Biotechnology, Dallas, TX, USA) was added followed by incubation at room temperature until the protein bands became visible.

### 2.4. Transmission Electron Microscopy (TEM)

The purified *Mr*NV-CP^JEV-DIII^ (0.25 to 0.30 mg/mL) was adsorbed to 300-mesh copper grids, and negatively stained with 2% (*w*/*v*) uranyl acetate for 8 min. The grids were then allowed to air-dry completely, and analyzed using a Hitachi H7700 transmission electron microscope (TEM, Hitachi, Tokyo, Japan).

### 2.5. Dynamic Light Scattering (DLS)

Purified VLPs were filtered with a 0.2 µm-syringe filter and transferred into the sample cell for analysis with the Zetasizer Nano ZS (Malvern, Worcestershire, UK). The hydrodynamic radius (R_h_) and polydispersity of the suspended VLPs were measured with a 25 mW solid state laser at a wavelength of 780 nm at 24 °C.

### 2.6. Antigenicity Assay

The antigenicity assay used in this study was modified from a previous study [21]. Purified *Mr*NV-CP^JEV-DIII^, *Mr*NV-CP and JEV-DIII were diluted to 0.2, 0.5, 1, 10, 20, 40, and 100 µg/mL in HEPES buffer (25 mM HEPES, 500 mM NaCl, pH 7.4). From each dilution, 100 µL of the purified proteins were coated on a 96-well microtiter plate and incubated at 4 °C overnight. The wells were washed three times with TBST buffer followed by blocking with 1× milk diluent (KPL, Milford, MA, USA; 200 µL) for 2 h with gentle agitation at room temperature. The plate was then washed again with TBST. Anti-JEV-DIII mAb (1:2000 dilution in TBS buffer; 100 μL) was then added, and incubated at room temperature for 2 h. Next, the wells were washed three times with TBST, and an anti-mouse secondary antibody conjugated to alkaline phosphatase (1:5000 dilution in TBS buffer; 100 μL) was added and incubated for 2 h at room temperature. The wells were then washed three times with TBST prior to addition of p-nitrophenyl phosphate (100 μL) into each well and incubated for 20 min. The absorbance A_406_ was measured with the ELx800 microtiter plate reader (BioTek, Winooski, VT, USA).

### 2.7. Immunization of Mice

All animal experiments were conducted in accordance with the guidelines of Institutional Animal Care and Use Committee (IACUC), Universiti Putra Malaysia (AUP number: R050/2019). Six-week-old female BALB/c mice (*n* = 8, each group) were acclimatized for 1 week. The mice were immunized subcutaneously with 100 µL of 0.34 mg/mL of (i) purified *Mr*NV-CP^JEV-DIII^ (group A), (ii) *Mr*NV-CP^JEV-DIII^ supplemented with alum (Thermo Scientific Imject Alum, Waltham, MA, USA) with 1:1 ratio (group B), (iii) *Mr*NV-CP (group C), (iv) *Mr*NV-CP supplemented alum (group D), (v) HEPES buffer, pH 7.4 (group E) and (vi) IMOJEV (Sanofi Pasteur, Bangkok, Thailand; 100 µL) (group F). The first injection was done on week 2 followed by administrations of the 1st and 2nd boosters on the 5th and 8th weeks, respectively. Approximately 100 µL blood were collected from each mouse via submandibular bleeding on the 2nd, 5th, and 8th weeks before each injection. Sera were harvested from blood samples via centrifugation at 1000× *g* for 10 min. The sera were kept at −80 °C prior to further analysis.

### 2.8. Immunogenicity Analysis

The immunogenicity of sera collected from immunized mice on the 2nd, 5th, 8th, and 9th weeks was analyzed with the enzyme-linked immunosorbent assay (ELISA). The JEV-DIII was expressed and purified as mentioned in Section 2.2. The concentration of purified JEV-DIII was determined by the Bradford assay [25]. The purified JEV-DIII was coated on a 96-well microtiter plate and incubated overnight at 4 °C. The wells were washed three times with TBST and blocked by 1x milk-diluent (KPL, Milford, MA, USA; 200 µL) for 1 h at room temperature. The wells were then washed with TBST as above, and 100 µL of 1:5000 diluted serum samples (diluted in TBS) were added and incubated for 2 h at room temperature. Afterwards, the wells were washed with TBST and alkaline phosphatase-conjugated anti-mouse mAb (1:5000 dilution in TBS, 100 µL) was added, followed by incubation for another 2 h at room temperature. Finally, the wells were washed three times with TBST, and p-nitrophenyl phosphate (100 µL per well) was added for color development. The plate was then incubated for 20 min in the dark, and the absorbance A_405_ was measured with the ELx800 microtiter plate reader (BioTek, Winooski, VT, USA).

### 2.9. Splenocyte Isolation and Flow Cytometry

Spleens were harvested from immunized mice a week after the second booster. The spleens were meshed through a 70 µm cell strainer in the presence of ice-cold PBS (Phosphate-buffered saline; 1 mL) to obtain a single cell suspension followed by centrifugation at 2000× *g* for 5 min at 4 °C. The cell pellet was resuspended gently with 5 mL chilled erythrocyte lysis buffer (10 mM KHCO_3_, 0.1 mM EDTA, 155 mM NH_4_Cl, pH 7.4) and the mixture was incubated on ice for 10 min. Approximately 6 mL of chilled PBS were added to the suspension followed by centrifugation at 2000× *g* for 5 min at 4 °C. The supernatant was discarded, and the cell pellet was resuspended in erythrocyte lysis buffer until a white pellet was observed. About 1 mL of chilled PBS containing 1% (*w*/*v*) BSA (bovine serum albumin) was added to resuspend the pellet gently. Approximately 5 × 10^6^ splenocytes were counted using a hemocytometer, and transferred into 1.5 mL tubes before antibodies were added into the tubes. All antibodies used in flow cytometry were purchased from Thermo Scientific, MA, USA. The phycoerythrin (PE) conjugated anti-CD4 mAb (0.125 µg), allophycocyanin (APC) conjugated anti-CD8 mAb (0.125 µg), fluorescein isothiocyanate (FITC) conjugated anti-CD69 + CD3 mAb (0.5 µg), FITC conjugated anti-CD3 mAb (0.25 µg), and FITC conjugated anti-F4/80 mAb (0.5 µg) were added to the splenocytes and incubated on ice away from light with agitation for 2 h. The antibody-stained splenocytes were washed with 0.5 mL of PBS and centrifuged at 2000× *g* for 5 min at 4 °C. Finally, the antibody-stained splenocytes were fixed with PBS buffer with 1% (*w*/*v*) paraformaldehyde and kept at 4 °C. Analysis of the splenocytes was carried out with a flow cytometer (BD FACSCanto II Flow Cytometer, Erembodegem, Belgium). Flow cytometry data analysis was performed using the BD FACSDiva™ software version 6.1.2 (BD Biosciences, San Jose, CA, USA).

### 2.10. Cytokine Quantification through Sandwich Enzyme-Linked Immunosorbent Assay

The concentrations of IL-6, IL-10, TNFα, and IFN-γ in mouse peripheral blood were determined using a multiplex ELISA array; Q-Plex™ Mouse Cytokine Panel 1 (Quansys Biosciences, Logan, UT, USA) and IL-12 p70 mouse ELISA Kit (Thermo Scientific, MA, USA). The quantification of IL-6, IL-10, IFN-γ, and TNFα was performed according to the manufacturer’s guideline using the Q-View™ Software systems (Quansys Biosciences, Logan, UT, USA). The IL-12p70 concentration was determined using the plate reader (TECAN Infinite M200R, Männedorf, Switzerland) at a wavelength of 450 nm.

### 2.11. Statistical Analysis

The variations of antigenicity of chimeric proteins, antibody titers, immunophenotyping, and cytokine concentrations were analyzed using one-way analysis of variance (ANOVA). Significant differences between groups were differentiated by the Duncan’s multiple-range method. A *p*-value less than 0.05 is considered significant and lesser than 0.001 is considered very significant. All data analysis was achieved using IBM SPSS statistics software version 22 (IBM Corporation, Armonk, NY, USA).

## 3. Results

### 3.1. Construction of Recombinant Plasmid, Protein Expression, and Purification

**Figure 1:**
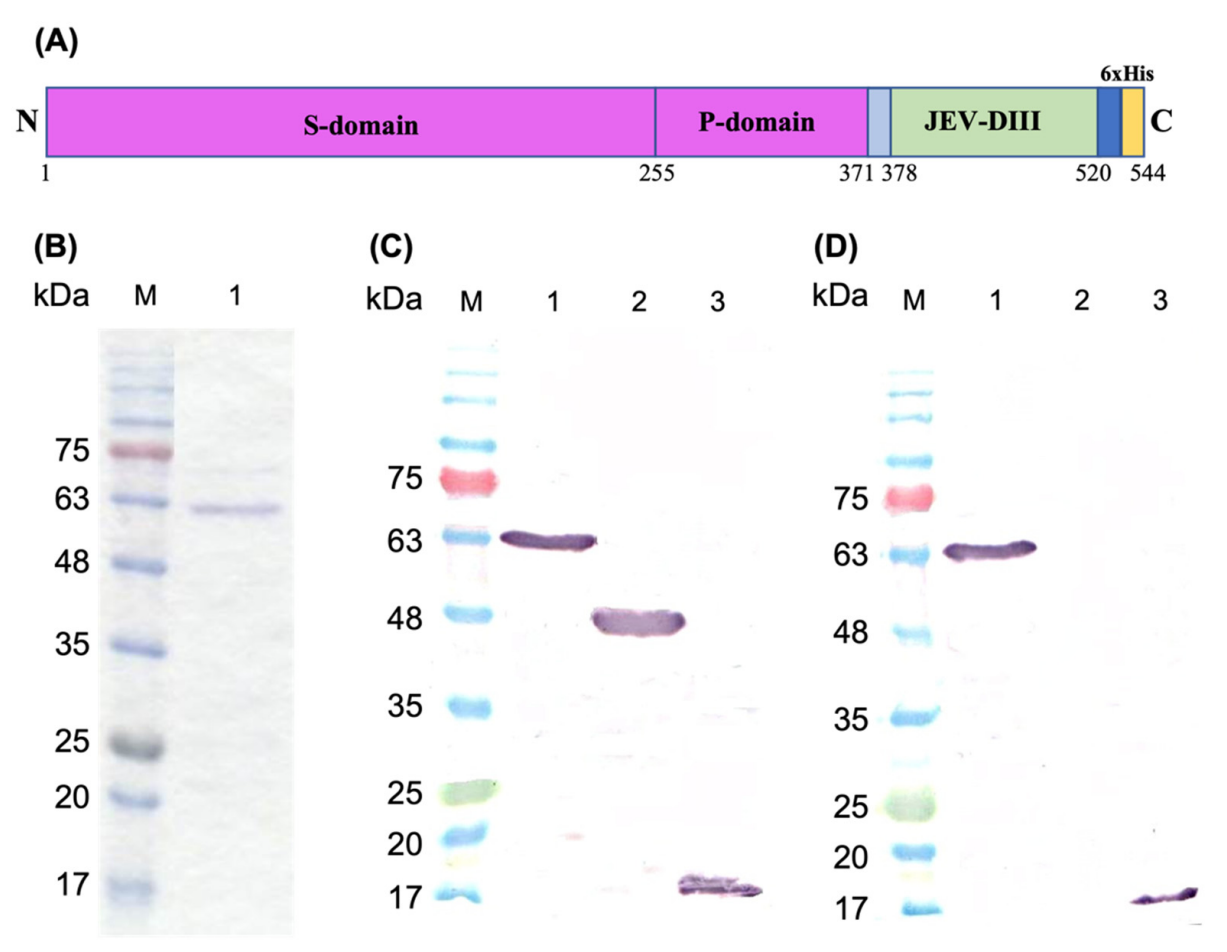
Schematic representation of recombinant protein, SDS-polyacrylamide gel electrophoresis and western blot analysis. (**A**) Schematic representation of recombinant *Macrobrachium rosenbergii* nodavirus capsid protein fused with domain III of Japanese encephalitis virus envelope protein (*Mr*NV-CP^JEV-DIII^). The amino acid positions of each domain in the fusion protein are indicated with numerical numbers. The amino- and carboxyl-termini are indicated as N and C, respectively. (**B**) Purification of *Mr*NV-CP^JEV-DIII^ with immobilized metal affinity chromatography (IMAC). Lane 1 indicates the protein eluted with 100 mM imidazole. In western blot analysis, purified proteins were probed with (**C**) anti-His monoclonal antibody, and (**D**) anti-JEV-DIII monoclonal antibody. Lanes 1, 2, and 3 are *Mr*NV-CP^JEV-DIII^, *Mr*NV-CP and JEV-DIII alone, respectively. Protein markers in kDa are indicated in each panel.

The coding region of JEV-DIII (344 bp) was successfully amplified from the reference strain of JEV (Nakayama) by one-step RT-PCR. The *Eco*RI and *Hin*dIII restriction enzyme cutting sites introduced in the primers were used to facilitate molecular cloning of JEV-DIII into pTrcHis-TARNA2 plasmid containing the full-length coding sequence of *Mr*NV-CP (Figure S1, Supplementary Materials). This recombinant plasmid encodes a fusion protein that comprises 544 amino acid residues including a myc and a 6x-His tag at the carboxyl terminus (Figure 1A). This recombinant plasmid was introduced into *E. coli* TOP10 host cells. Protein expression was achieved by IPTG induction at 30 °C for 5 h. The recombinant protein was purified using IMAC, which was eluted as a protein of ~60 kDa (Figure 1B). The size of this protein corresponds well with the calculated molecular mass of *Mr*NV-CP^JEV-DIII^ (60,218.11 Da). Figure 1C shows that *Mr*NV-CP^JEV-DIII^, *Mr*NV and JEV-DIII, each containing a 6x-His tag, were detected by anti-His mAb with molecular masses of ~60 kDa, ~45 kDa, and ~17 kDa, respectively. Similarly, both recombinant proteins, *Mr*NV-CP^JEV-DIII^ and JEV-DIII, containing the domain III of JEV E protein were also detected by anti-JEV-DIII mAb (Figure 1D).

### 3.2. Transmission Electron Microscopy

**Figure 2:**
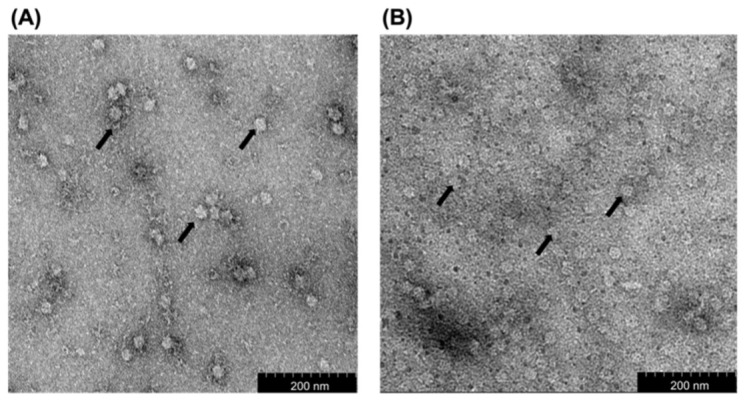
Transmission electron micrographs of the purified *Macrobrachium rosenbergii* nodavirus capsid protein (*Mr*NV-CP) and its fusion counterpart *Mr*NV-CP^JEV-DIII^. Micrographs (**A**,**B**) show that the *Mr*NV-CP and *Mr*NV-CP^JEV-DIII^ assembled into spherical VLPs (arrows) with diameters ~30 nm and ~18 nm, respectively. The purified protein samples were stained with 2% (*w*/*v*) of uranyl acetate and viewed under 200,000× magnification. Scale bars are indicated in the micrographs.

TEM analysis showed that the diameter of VLPs derived from the purified *Mr*NV-CP and *Mr*NV-CP^JEV-DIII^ was ~30 and ~18 nm, respectively (Figure 2). Interestingly, fusion of JEV-DIII at the C-terminal end of *Mr*NV-CP caused a reduction in the diameter of the VLPs by ~12 nm compared to its native counterpart.

### 3.3. Dynamic Light Scattering (DLS) Analysis

**Figure 3:**
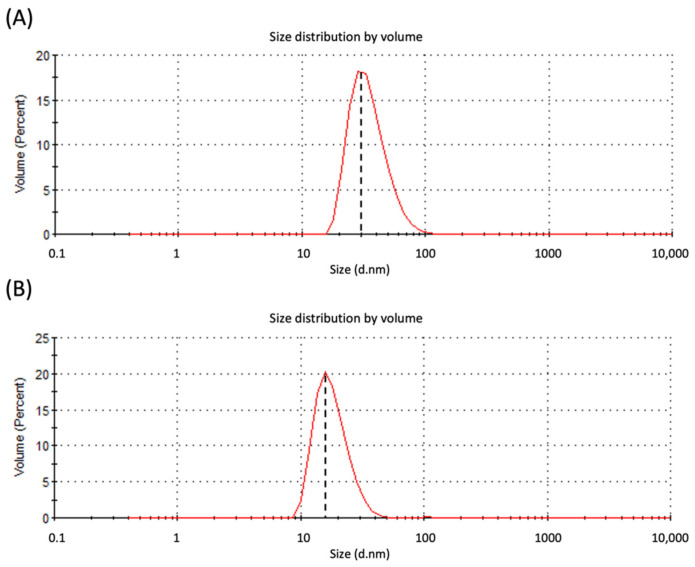
Dynamic light scattering (DLS) analysis of the purified *Macrobrachium rosenbergii* nodavirus capsid protein (*Mr*NV-CP) and its fusion counterpart *Mr*NV-CP^JEV-DIII^. The vertical dotted lines intersecting the peaks (**A**,**B**) represent the mean diameter of the virus-like particles (VLPs) derived from the full-length *Macrobrachium rosenbergii* nodavirus capsid protein (*Mr*NV-CP) and *Mr*NV-CP fused with domain III of Japanese encephalitis virus envelope protein (*Mr*NV-CP^JEV-DIII^)*,* respectively.

Dynamic light scattering (DLS) was performed to determine the hydrodynamic radius and polydispersity of the VLPs. In this study, DLS analysis revealed that the average particulate size of VLPs derived from the full-length *Mr*NV-CP and *Mr*NV-CP^JEV-DIII^ was 35.5 nm and 18.2 nm, respectively (Figure 3). The polydispersity index for VLPs derived from the full-length *Mr*NV-CP and *Mr*NV-CP^JEV-DIII^ was 11.6% and 58.5%, respectively.

### 3.4. Analysis of the Antigenicity of the Fusion Protein by Enzyme-Linked Immunosorbent Assay (ELISA)

**Figure 4:**
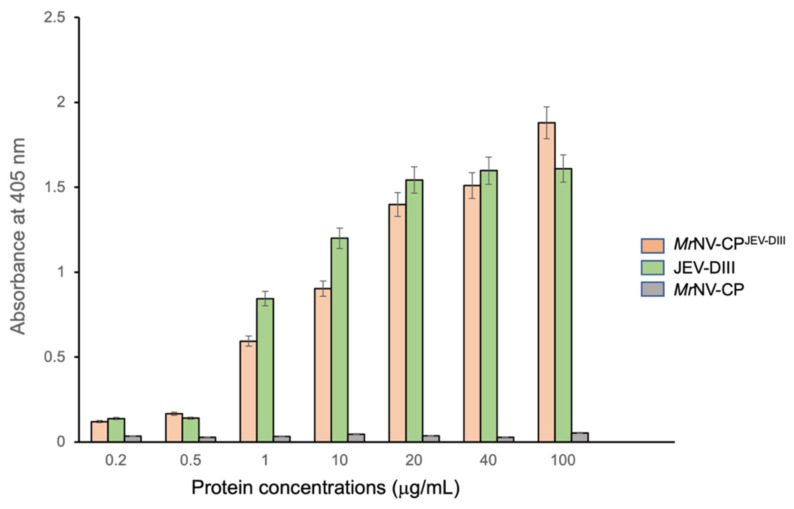
Antigenicity of the domain III of Japanese encephalitis virus envelope protein fused at the C-terminus of *Macrobrachium rosenbergii* nodavirus capsid protein (*Mr*NV-CP^JEV-DIII^). *Mr*NV-CP was used as a negative control and JEV-DIII alone was used as a positive control. Error bars indicate standard deviations of triplicate measurements.

ELISA was performed to study the antigenicity of *Mr*NV-CP^JEV-DIII^ in its native state. The chimeric protein was diluted to 0.2, 0.5, 1, 10, 20, 40, and 100 µg/mL and coated on a 96-well microtiter plate. The *Mr*NV-CP and JEV-DIII were used as negative and positive controls, respectively. Figure 4 shows that the antigenicity of *Mr*NV-CP^JEV-DIII^ and JEV-DIII increased slowly at low protein concentrations (0.2 and 0.5 µg/mL), followed by a sharp, approximately four- and six-fold rise of antibody titers, respectively, when the concentration of the protein was increased to 1 µg/mL. The antigenicity of *Mr*NV-CP^JEV-DIII^ continued to increase proportionally to protein concentrations. Interestingly, the antigenicity of *Mr*NV-CP^JEV-DIII^ was not plateaued at its highest concentration (100 µg/mL) whereas the antigenicity of JEV-DIII was leveled off at 20 µg/mL. At the highest protein concentration, the antigenicity of *Mr*NV-CP^JEV-DIII^ was ~16.5% higher than that of JEV-DIII. The negative control (*Mr*NV-CP) was not detected by anti-JEV-DIII mAb. This result demonstrated that the VLPs of *Mr*NV-CP^JEV-DIII^ were antigenic, comparable to the positive control, JEV-DIII.

### 3.5. Immunogenicity of the Fusion Protein

**Figure 5:**
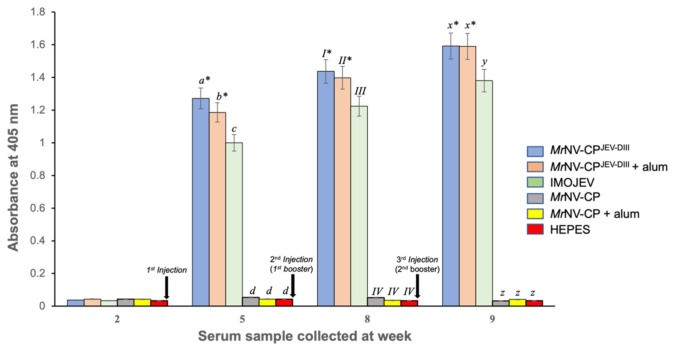
Immunogenicity of the domain III of Japanese encephalitis virus envelope protein (JEV-DIII) in BALB/c mice. Total antibodies against JEV-DIII in serum samples collected at week 2 (before first injection), week 5 (before first booster), week 8 (before second booster), and week 9 (one week after second booster). The JEV-DIII protein was coated onto the microtiter plate wells and reacted with serum antibodies collected from mice injected with *Mr*NV-CP^JEV-DIII^ supplemented with or without alum, IMOJEV, *Mr*NV-CP with or without alum, and HEPES. Alphabets (*a*, *b*, *c* and *d*; *x*, *y* and *z*) and Roman numerals (*I*, *II*, *III* and *IV*) on top of each bar denote statistical significance (*p* < 0.001) within each time point. Same alphabets or Roman numerals indicate insignificant difference. Asterisks (*) on top of the bar charts show significant difference (*p* < 0.001) compared with the group of mice immunized with IMOJEV (positive control). The error bars indicate standard deviations of triplicate measurements.

After the mice were acclimatized for one week, they were immunized subcutaneously at week 2 (first injection), week 5 (first booster), and week 8 (second booster) with *Mr*NV-CP and *Mr*NV-CP^JEV-DIII^ with or without adjuvant. IMOJEV and HEPES were used as the positive and negative controls, respectively. Blood samples were collected before each injection and antibodies against the JEV-DIII were determined by ELISA. Figure 5 indicates that no antibody against JEV-DIII was detected in the mouse sera prior to immunization. Significant amounts of antibody against JEV-DIII were produced after three weeks of the first injection with *Mr*NV-CP^JEV-DIII^ with or without adjuvant and IMOJEV compared to the buffer immunized group. Intriguingly, non-adjuvanted *Mr*NV-CP^JEV-DIII^ elicited a significantly higher JEV-DIII-specific antibody titer compared to those immunized with *Mr*NV-CP^JEV-DIII^ adjuvanted with alum or IMOJEV. Subsequent boosters at week 5 and 8 further increased the antibody titers of *Mr*NV-CP^JEV-DIII^ adjuvanted with alum, or IMOJEV. Mice immunized with IMOJEV exhibited a consistently lower JEV-DIII-specific antibody titer throughout the immunization period compared to those administered with *Mr*NV-CP^JEV-DIII^ regardless of the presence of adjuvant. All mice immunized with *Mr*NV-CP lacking JEV-DIII failed to elicit significant JEV-DIII-specific antibody response. JEV-DIII-specific antibody remained undetected in sera collected from mice inoculated with *Mr*NV-CP regardless of the presence of adjuvant, or HEPES buffer.

### 3.6. Immunophenotyping of Mouse Splenocytes

**Figure 6:**
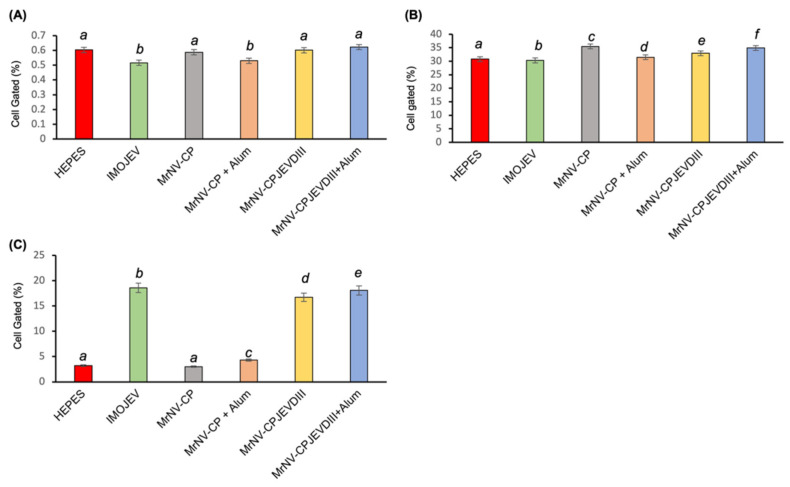
Immunophenotyping of mouse splenocytes. Frequency of (**A**) CD8^+^/CD4^+^ ratios, (**B**) natural killer (NK) cells, and (**C**) macrophages isolated from mouse splenocytes. The anti-CD4 monoclonal antibody labelled with R-phycoerythrin (PE), anti-CD8 monoclonal antibody labelled with allophycocyanin (APC), anti-CD69 + CD3 monoclonal antibody labelled with fluorescein isothiocyanate (FITC) and anti-F4/80 monoclonal antibody labelled with FITC, were added to the 5 × 10^6^ splenocytes and analyzed with a flow cytometer. The letters (*a*, *b*, *c*, *d*, *e* and *f*) on top of the bars represent the statistical significance of measurements. Different letters indicate significant difference (*p* < 0.001), same letters are not significantly different. The error bars represent the standard deviations of triplicate measurements.

The spleens of immunized mice were collected a week after the second booster (week 9). The purpose of harvesting the spleens was to study the presence of natural killer (NK) cells, cytotoxic T lymphocytes (CTL), and helper T lymphocytes (Th) via immunophenotyping. Table 1 shows that the populations of CD3^+^CD4^+^ and CD3^+^CD8^+^ T cells are higher in splenocytes isolated from mice injected with *Mr*NV-CP^JEV-DIII^ with or without adjuvant, *Mr*NV-CP and IMOJEV compared to the mocked immunized group. Nevertheless, the CD8/CD4 T-cell ratio was reported to be not significantly altered in those immunized with *Mr*NV-CP or *Mr*NV-CP^JEV-DIII^ regardless of the presence of adjuvant compared to the mock-immunized group (Figure 6A). IMOJEV also induced a significant increase in the CD3^+^CD4^+^ population compared to the buffer group. However, IMOJEV induced a lower CD8/CD4 T-cell ratio, suggesting a CD4-T-cell dominant response. Similar profiles were also observed for NK cell population in mice immunized with recombinant proteins with or without adjuvant (Figure 6B). Intriguingly, mice immunized with IMOJEV and *Mr*NV-CP^JEV-DIII^ with or without adjuvants demonstrated significantly higher levels of macrophage populations compared to the negative control groups and *Mr*NV-CP with or without adjuvant (Figure 6C), suggesting a critical role of JEV-DIII in induction of macrophage expansion or proliferation of splenic macrophage.

### 3.7. Measurement of Cytokine Concentrations in Mouse Serum Samples

**Figure 7:**
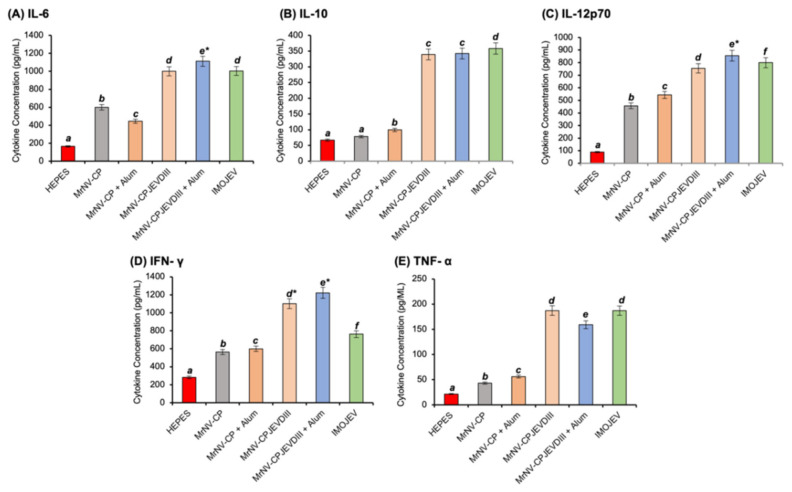
Multiplex quantification of cytokines in sera of immunized mice. Different types of cytokines were quantitated from sera collected from the negative control group (HEPES, *Mr*NV-CP with and without alum), test group (*Mr*NV-CP^JEV-DIII^ with and without alum), and positive control group (IMOJEV). The letters (*a*, *b*, *c*, *d*, *e*, *f)* shown above the bars represent statistical significance of the measurements. Different letters represent significant difference (*p* < 0.001), and the same letters are not significantly different. Asterisks indicate *p* < 0.001, comparing the cytokine concentrations of the test group with the positive control groups (IMOJEV) of this study. The error bars denote the mean (±) standard deviation of triplicate measurements.

A total of five cytokines were analyzed in this study, IL-6, IL-10, IL-12p70, IFN-γ, and TNFα (Figure 7). In general, all mice immunized with the recombinant proteins regardless of the presence of adjuvant, and IMOJEV demonstrated a significant increase in all serum cytokines tested. Nevertheless, a stronger magnitude was observed in mice immunized with IMOJEV and *Mr*NV-CP^JEV-DIII^ than those that received *Mr*NV-CP and buffer groups. Mice immunized with *Mr*NV-CP^JEV-DIII^ regardless of the presence of adjuvant elicited high titers of IFN-γ compared to mice immunized with IMOJEV. High levels of IL-10 were also noticed for mice administered with *Mr*NV-CP^JEV-DIII^ (with or without adjuvant) and the positive control, IMOJEV compared to the *Mr*NV-CP and buffer groups. Interestingly, the profile of TNF-α is similar to IL-10, except a slightly lower titer in mice administered with *Mr*NV-CP^JEV-DIII^ in the presence of an adjuvant.

## 4. Discussion

To date, there is no effective anti-JE drug available to treat the disease. As eradication of vectors (mosquito) is impossible, vaccination remains the only strategy to prevent humans from JEV infection. The first JE vaccine was developed from mouse brain-derived inactivated (MBDI) vaccine. Despite the effectiveness of MBDI vaccine in reducing the number of JE cases in some endemic countries, its production was halted due to its adverse side-effects such as hypersensitivity and neurological complications [26,27,28]. Newer JE vaccines include chimeric virus, inactivated Vero cell culture vaccine based on SA14-14-2 strain, and live attenuated chimeric vaccine based on combination of yellow fever 17D (YF17D) vaccine strain and Vero cell-propagated SA-14-14-2 strain cultured in primary hamster kidney cells (IMOJEV). Although the recombinant live vaccines have been proven safe and protective, they are not recommended for immunocompromised individuals, pregnant and lactating women [29]. Furthermore, the major inherent risk of using these live-attenuated vaccines is the reversion to virulence in live-attenuated vaccine viruses. Therefore, development of novel JE vaccines with higher safety profile and protectivity is required.

In this study, we displayed the JEV-DIII on the outer surface of the VLPs of *Mr*NV-CP^JEV-DIII^. Our results showed that *Mr*NV-CP^JEV-DIII^ was successfully expressed and purified as an ~60 kDa protein as analyzed with SDS-PAGE. Meanwhile, western blot analysis showed that *M*rNV-CP^JEV-DIII^ can be detected by anti-JEV-DIII mAb. TEM analysis revealed that the diameter of the VLPs derived from *Mr*NV-CP^JEV-DIII^ was significantly smaller than that assembled from the full-length *Mr*NV-CP. Previous studies showed that insertion of foreign epitopes ~49 amino acid residues at the C-terminus of *Mr*NV-CP did not significantly change the diameter of the VLPs compared to those formed by the wild-type protein when they were expressed in *E. coli* [21,22]. However, fusion of 170 amino acid residues; inclusive of JEV-DIII (135 residues) and 6xHis tag, at the C-terminal end of *Mr*NV-CP, has reduced the diameter of the VLPs to ~18 nm, approximately 12 nm smaller than its native counterpart. It is believed that the addition of such a large protein fragment has caused conformational changes particularly to the protruding (P) domain at the C-terminal region of *Mr*NV-CP [30,31]. *Mr*NV-CP^JEV-DIII^ may assemble to form icosahedral structures with triangulation number *T* = 1 instead of *T* = 3 as observed in the native *Mr*NV-CP. It is likely that the asymmetric units had been rearranged to accommodate the large fusion polypeptide at the C-terminal region. DLS analysis showed that the full length *Mr*NV-CP and recombinant *Mr*NV-CP^JEV-DIII^ formed VLPs with average diameters of 35.5 nm and 18.2 nm, respectively. The smaller particles formed by the *Mr*NV-CP^JEV-DIII^, however, were less homogenous compared to those of wild-type *Mr*NV-CP. This is most likely due to the intrinsic stability of the wild-type protein compared to the recombinant counterparts. Similar to the observation by Ninyio et al. [19], the hydrodynamic radius of the chimeric particles displaying hepatitis B virus’ ‘a’ determinant as analyzed with DLS was slightly larger, compared to the measurement determined by TEM. The major difference in diameter between DLS and TEM analyses could be due to DLS measurement of hydrodynamic radius of particles was performed in solution while in TEM, the diameter was measured on a dried carbon grid [32]. Furthermore, the diameter of particulates determined by DLS is also affected by ions associated with viral particles [33]. Some studies showed that changes in virion morphology and stability are related to the number of cysteine residues present in the foreign peptide, which suggests additional cysteine disulfide bonds could affect capsid formation, oligomerization, and capsid stabilization [34,35,36,37]. The JEV-DIII consists of two cysteine residues at positions 13 and 44. However, it remains elusive how these cysteine residues might affect the formation of VLPs in this study. Elucidation of the three-dimensional structure of *Mr*NV-CP^JEV-DIII^ derived VLPs is essential to reveal the molecular details of the *Mr*NV-CP with the P-domain fused with JEV-DIII.

Alteration of VLPs’ diameter, however, did not jeopardize the antigenicity of the chimeric VLPs. The VLPs of *Mr*NV-CP^JEV-DIII^ and the positive control (JEV-DIII alone) coated on the microtiter plates were shown to be detected by the anti-JEV-DIII mAb, suggesting that the JEV-DIII epitopes were exposed on the VLPs surface. In addition, the antigenicity exhibited by the chimeric VLPs is comparable to that of JEV-DIII alone, indicating that fusion of the JEV-DIII to the capsid protein did not impose significant steric hindrance on antibody affinity. Similar observations were also reported when the antigenic determinant of hepatitis B virus and the extracellular domain of the M2 protein of influenza A virus were displayed on the surface of *Mr*NV-CP VLPs [19,21,22].

Antibody responses induced by JEV-DIII in BALB/c mice were determined by ELISA. Our results showed that antibody specific against JEV-DIII was elicited in mice injected with *Mr*NV-CP^JEV-DIII^ regardless of the presence of adjuvant as observed in week 5. Although the presence of adjuvant could slightly increase antibody production at weeks 5 and 8 in mice vaccinated with *Mr*NV-CP^JEV-DIII^, its function in enhancing antibody titer was not significant. This result deviates from previous studies employing a similar immunization strategy and nodavirus carrier, but displaying different foreign epitopes, of which no significant antibody response following primary injection was observed unless an adjuvant was incorporated in the vaccine formulation [20,21,22]. Two booster injections further elevate the antibody responses of the mice immunized with *Mr*NV-CP^JEV-DIII^, and IMOJEV. Our results also indicate that addition of an adjuvant is dispensable, at least in the mouse model used in this study. In fact, VLPs have been reported to function as vaccine antigens and also as adjuvants [38]. Gilbert [39] showed that VLPs displayed their adjuvanticity when they were taken up by antigen presenting cells (APC), and the peptides derived from them were presented on MHC class I molecules on the cell surface, and subsequently primed CD8^+^ T cell responses.

Fusion proteins comprising Th epitopes have been shown to enhance the antibody production against the B cell determinant [40], and fusion of a Th epitope to a CTL determinant led to the generation of antiviral CTLs [41]. Furthermore, the Th cells triggered by a vaccine carrying CD4^+^ Th epitopes have been demonstrated to secrete numerous CTL-inducing or antiviral cytokines, stimulate CTL responses, and preserve CTL memory [42]. The domain III of JEV is the viral major immunogenic antigen of the virus as it contains various T- and B-cell epitopes including selective epitopes of Th (residues 436–445), and epitopes of B-cell (residues 356–362, 373–399 and 397–401) [43]. JEV-DIII specific CD4 and CD8 T-cells are critical for mediating protection against JEV. CD4 T-cells facilitate development of B-cells and CD8 T-cells promote viral clearance [41]. The immunophenotyping results of this study demonstrated that the BALB/c mice immunized with *Mr*NV-CP^JEV-DIII^ with or without alum showed significant higher ratios of CD8^+^/CD4^+^ (CTL to Th) as compared to that of IMOJEV, but insignificant if compared to that of *Mr*NV-CP. However, both CD3^+^CD4^+^ and CD3^+^CD8^+^ T cell populations increased significantly following immunization with *Mr*NV-CP^JEV-DIII^ VLPs compared to the buffer group, demonstrating the capability of JEV-DIII in inducing the expansion of T cells. Activation of T cells is important in preventing breach in the blood brain barrier (BBB), which leads to encephalitis [42]. Although both CD4^+^ and CD8^+^ T cells were shown to be involved in protection against JEV infection, depletion of CD4+ severely affected the survival rate of mice compared to depletion of CD8^+^ T-cells [44]. However, a recent study proposed that CD8^+^ T-cells could play a role in primary infection and inhibiting the opening of BBB and neuronal complication during JEV infection [42].

Macrophages play a vital role in inflammatory responses and innate immunity during primary JEV infection by releasing soluble factors which induce neuro-apoptosis or necrosis [45]. Intriguingly, all mice immunized with *Mr*NV-CP^JEV-DIII^ induced a higher level of macrophage but not those immunized with *Mr*NV-CP, suggesting that the observed responses were JEV-DIII dependent. Mice immunized with *Mr*NV-CP^JEV-DIII^ (non-replicative VLPs) showed almost a similar level of macrophages to the live vaccine IMOJEV. The increase of macrophages in VLPs immunized mice could be related to the expression of activation marker CD80, a co-stimulatory molecule displayed on the surface of antigen presenting cells [46].

Natural killer (NK) cells are also known as the first line of cytotoxic defense mechanism as they are responding to primary infection and involved in early control of virus infection [47]. Mice immunized with VLPs have been shown to increase the NK cell count in the lymph nodes and spleens, which led to the secretion of TNF-α and IFN-γ and activated cytotoxic activity [48]. In this study, NK cell counts were noticed slightly higher in mice immunized with *Mr*NV-CP, *Mr*NV-CP^JEV-DIII^ with and without adjuvant compared to other groups, indicating that these VLPs are capable to activate the NK cells in vivo. Specifically, the NK cells do not reorganize antigen receptors to permit antigen-specific recognition; it is the innate immune system which recognizes the VLPs as an antigen [47]. Unlike T and B cells, NK cells which lack antigen-specific receptors are believed to destroy tumor cells without prior sensitization and mediate cytolysis in an unspecific fashion [49]. Consequently, the functions of NK cells have been modulated to enhance vaccine success due to their capacity to regulate adaptive immune responses, generate a unique form of innate immune memory, and functionally cooperate with vaccine-elicited components such as antibodies to prevent virus infections [50].

Several studies showed that numerous pro-inflammatory mediators such as TNF, IFN-α, RANTES, IL-6, and IL-8 are linked with the severity of JE [51,52,53]. IL-6 plays a role in host defense against infectious diseases [54] while IL-12 is naturally produced by macrophages, neutrophils, dendritic cells, and B-lymphoblastoid cells [55]. IL-12 induces production of Th1, which subsequently triggers the production of IFN-γ, which in turn, provides a defense mechanism via mediated antiviral protection, macrophage activation, Th1/Th2 balance regulation, and cellular proliferation [56]. The ability of vaccine candidates to induce a sufficient level of cytokines is crucial for the activation of the host’s innate immune response. Our results showed that *Mr*NV-CP^JEV-DIII^ induced cytokine responses comparable to that of the IMOJEV group. As shown in this study, *Mr*NV-CP alone regardless of the presence of adjuvant induced low levels of cytokines as compared to *Mr*NV-CP displaying the JEV-DIII with or without adjuvant. Our result implied that the significant elevation of the cytokines levels was attributed to the JEV-DIII displayed on the surface of the VLPs. Both Th1 (IFN-γ, IL-12p70, TNF-α) and Th2 cytokines (IL-6, IL-10) were increased concomitantly, suggesting a balance Th1/Th2 response induced by IMOJEV and *Mr*NV-CP^JEV-DIII^ with or without adjuvants. Th1 cytokines were known to be classical proinflammatory mediators which promote inflammation and viral clearance and IL-12 was known to drive IFN-γ secretion which exhibits direct antiviral effects [57]. On the other hand, Th2 cytokines act antagonistically against Th1 responses [57]. For instance, IL-10 was well known as an anti-inflammatory cytokine, of which reduced expression was demonstrated to correlate with disease severity in JEV infection [58]. Overall, our results indicated that the mice immunized with *Mr*NV-CP^JEV-DIII^ in the presence or absence of adjuvant could elicit innate and adaptive cellular immune responses.

## 5. Conclusions

In conclusion, we have demonstrated that the *Mr*NV-CP^JEV-DIII^ VLPs expressed in *E. coli* were immunogenic, regardless of the presence of alum as an adjuvant. The immunogenicity of *Mr*NV-CP^JEV-DIII^ with or without adjuvant was demonstrated to be comparable, if not stronger than that triggered by IMOJEV. These chimeric VLPs induced high levels of antibodies against JEV DIII. Immunophenotyping showed that mice immunized with *Mr*NV-CP^JEV-DIII^ regardless of the presence of adjuvant triggered the production of macrophages, NK cells, CTLs, and cytokines. These results confirmed that the *Mr*NV-CP^JEV-DIII^ VLPs may serve as an alternative approach as a vaccine candidate against JEV infections. However, the detailed protective mechanism, the effects of different adjuvants, and animal virus challenge in immunized animals need to be studied intensively before this vaccine candidate can enter a clinical trial.

## 6. Patents

A patent entitled “A virus-like particles based vaccine for Japanese encephalitis virus (JEV)” (Patent application no: PI2020005529) was filed on 21 October 2020.

## Figures and Tables

**Table 1:** Immunophenotyping of mice splenocytes.

Groups	Percentage of Cell Gated (%)	
	CD3^+^CD4^+^	CD3^+^CD8^+^
HEPES	14.01 ± 0.41 *a*	8.56 ± 0.15 *v*
IMOJEV	16.80 ± 0.55 *b*	8.70 ± 0.49 *w*
*Mr*NV-CP	17.03 ± 0.44 *c*	10.11 ± 0.58 *x*
*Mr*NV-CP with alum	14.41 ± 0.40 *d*	7.67 ± 0.15 *y*
*Mr*NV-CP^JEV-DIII^	18.20 ± 0.1 *e*	11.00 ± 0.36 *z*
*Mr*NV-CP^JEV-DIII^ with alum	17.70 ± 0.56 *f*	11.06 ± 0.58 *z*

All data were tested for statistical significances (*p* < 0.001). Immunophenotyping of mice (splenocytes) injected with HEPES, IMOJEV vaccine, *Mr*NV-CP supplemented with and without alum, *Mr*NV-CP^JEV-DIII^ with and without alum was performed and tabulated. CD3^+^CD4^+^ indicates the percentage population of Th cells in overall splenocytes, whereas CD3^+^CD8^+^ indicates CTLs. Different small letters represent significant difference (*p* < 0.001), and the same letters are not significantly different.

## Data Availability

The data presented in this study are available in Immunological Investigation of the Domain III of Japanese Encephalitis Virus Envelope Protein Displayed on the Virus Like Particles of *Macrobrachium rosenbergii* Nodavirus Capsid.

## Supplementary Materials

The following are available online at https://www.mdpi.com/article/10.3390/pharmaceutics13111826/s1, Figure S1: Construction of recombinant plasmid encoding the fusion protein of *Macrobrachium rosenbergii* nodavirus capsid protein (*Mr*NV-CP) and the domain III of Japanese encephalitis virus envelope protein (JEV-DIII), Raw data: Western blot analysis.
