# Supplementary material for: Immunological Analysis of Nodavirus Capsid Displaying the Domain III of Japanese Encephalitis Virus Envelope Protein
Source: Pharmaceutics. 2021 Nov 1;13(11):1826. doi: 10.3390/pharmaceutics13111826 (PMC8618745; doi:10.3390/pharmaceutics13111826)
Supplement: Supplementary file 1 [file pharmaceutics-13-01826-s001.zip › pharmaceutics-1395410-supplementary.pdf]

pharmaceutics

MDPI

Supplementary Materials: Immunological Analysis of Nodavirus Capsid Displaying the Domain III of Japanese Encephalitis Virus Envelope Protein

Kiven Kumar, Hui Kian Ong, Wen Siang Tan, Siti Suri Arshad, Kok Lian Ho

(A)

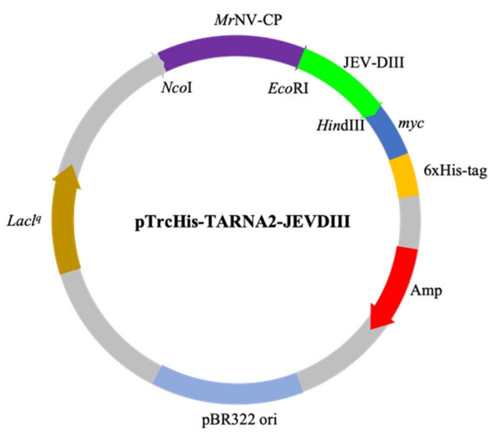

(B)

```
        10         20         30         40         50         60
MALNIKMARG KQNSNQAQNN SNANGKRRKR SRRNRNPQTI PNFNPIVAKP TVAPLQTNIR

        70         80         90        100        110        120
SARSDVNAIT VLNGSDFLTT VKVRGSNNLT DSKSRILVKQ PISASSFLGT RISGLSQFWE

       130        140        150        160        170        180
RYRWHKAAVR YVPAVPNTLA CQLIGYIDTD PLDDPNVILD VDQLLRQATS QVGARQWNFS

       190        200        210        220        230        240
DTTTIPLIVR RDDQLYYTGQ DKENVRFSQQ GVFYLLQVTT LLNISGEAIT NDLISGSLYL

       250        260        270        280        290        300
DWVCGFSMPQ INPTPVEISQ LTYNADTIGN WVPPTELNQT YTQDITGLKP NSKFIIVPYM

       310        320        330        340        350        360
DRTSSEVLQK CTITCNEVNA VGSISYFDTN DIKCNGYITF QANNIGEATF TLVTDYKGVT

       370        380        390        400        410        420
DPKPYQYRII RAIVGNNKGE FGGGGWSHLK CRLRMDKLAL KGTTYGMCTE KFSFAKNPAD

       430        440        450        460        470        480
TGHGTVVIEL SYSGSDGPCK IPIVSVASLN DMTPVGRLVT VNPFVATSSA NSKVLVMEPP

       490        500        510        520        530        540
FGDSYIVVGR GDKQINHHWH KAGSTLGKAF STTLKGAQTQ AYVEQKLISE EDLNSAVDHH

HHHH
```

Figure S1. Construction of recombinant plasmid encoding the fusion protein of *Macrobrachium rosenbergii nodavirus* capsid protein (*Mr*NV-CP) and the domain III of Japanese encephalitis virus envelope protein (JEV-DIII). **A**) A map of recombinant plasmid harboring the coding sequence of *Mr*NV-CP (purple) fused with the coding sequence of the JEV-DIII (green) at its 3′ end, followed by the myc (dark blue) and 6xHis-tag (yellow) nucleotide sequences. **B**) The primary amino acid sequence of the fusion protein *Mr*NV-CP$^{JEV\text{-}DIII}$ is colored as described above. Amino acid residues colored in light blue are additional amino acids from the envelope protein.

RAW DATA

Western blot analysis

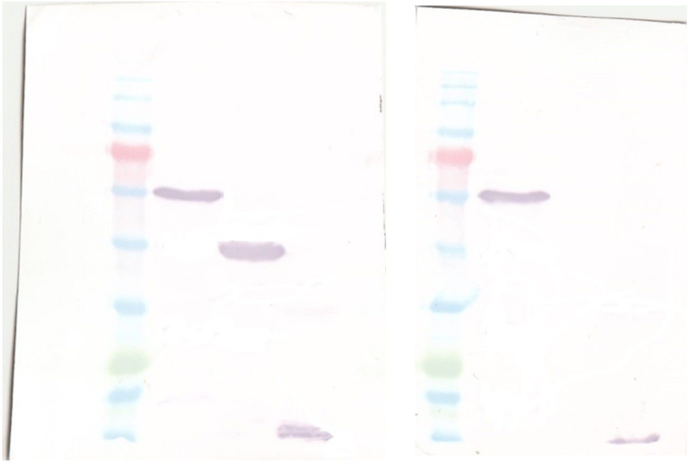

(**a**) (**b**)

In western blot analysis, purified proteins were probed with (**a**) anti-His monoclonal antibody, and (**b**) anti-JEV DIII monoclonal antibody**.** Lanes 1, 2 and 3 are *Mr*NV-CP$^{JEV\text{-}DIII}$, *Mr*NV-CP and JEV-DIII alone, respectively. Protein markers in kDa are indicated in each panel.
